# Functional gradients reveal cortical hierarchy changes in multiple sclerosis

**DOI:** 10.1002/hbm.26678

**Published:** 2024-04-22

**Authors:** Alessandro Pasquale De Rosa, Alessandro d'Ambrosio, Alvino Bisecco, Manuela Altieri, Mario Cirillo, Antonio Gallo, Fabrizio Esposito

**Affiliations:** ^1^ Advanced MRI Neuroimaging Centre, Department of Advanced Medical and Surgical Sciences University of Campania “Luigi Vanvitelli” Naples Italy

**Keywords:** functional connectivity, functional gradients, machine learning, multiple sclerosis, resting‐state fMRI

## Abstract

Functional gradient (FG) analysis represents an increasingly popular methodological perspective for investigating brain hierarchical organization but whether and how network hierarchy changes concomitant with functional connectivity alterations in multiple sclerosis (MS) has remained elusive. Here, we analyzed FG components to uncover possible alterations in cortical hierarchy using resting‐state functional MRI (rs‐fMRI) data acquired in 122 MS patients and 97 healthy control (HC) subjects. Cortical hierarchy was assessed by deriving regional FG scores from rs‐fMRI connectivity matrices using a functional parcellation of the cerebral cortex. The FG analysis identified a primary (visual‐to‐sensorimotor) and a secondary (sensory‐to‐transmodal) component. Results showed a significant alteration in cortical hierarchy as indexed by regional changes in FG scores in MS patients within the sensorimotor network and a compression (i.e., a reduced standard deviation across all cortical parcels) of the sensory‐transmodal gradient axis, suggesting disrupted segregation between sensory and cognitive processing. Moreover, FG scores within limbic and default mode networks were significantly correlated (ρ=0.30, *p* < .005 after Bonferroni correction for both) with the symbol digit modality test (SDMT) score, a measure of information processing speed commonly used in MS neuropsychological assessments. Finally, leveraging supervised machine learning, we tested the predictive value of network‐level FG features, highlighting the prominent role of the FG scores within the default mode network in the accurate prediction of SDMT scores in MS patients (average mean absolute error of 1.22 ± 0.07 points on a hold‐out set of 24 patients). Our work provides a comprehensive evaluation of FG alterations in MS, shedding light on the hierarchical organization of the MS brain and suggesting that FG connectivity analysis can be regarded as a valuable approach in rs‐fMRI studies across different MS populations.

## Graphical abstract

- Functional connectivity gradients reveal significant alterations in the sensorimotor network hierarchy and a compression of the sensory‐transmodal gradient, indicating disrupted segregation between sensory and cognitive processing, in multiple sclerosis patients.
- Functional connectivity gradients within limbic and default mode networks are associated with information processing speed in multiple sclerosis patients.

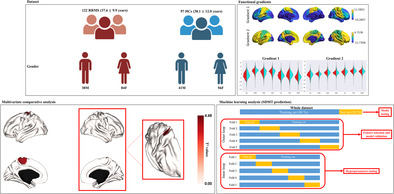

## 1 INTRODUCTION

Multiple sclerosis (MS) is a neuroinflammatory and neurodegenerative disease of the central nervous system (Filippi et al., 2018). The clinical manifestations of MS are heterogeneous, encompassing a wide range of symptoms, from physical (e.g., visual, sensory and motor disturbances) to cognitive impairments (e.g., deficits in information processing speed [IPS], episodic memory, attention, executive function) (Filippi et al., 2018). Since MS can be regarded as a brain network disorder (Schoonheim et al., 2022), many studies have explored the impact of the MS disease on functional connectivity (FC) estimated from resting‐state functional MRI (rs‐fMRI) time‐series (Bisecco et al., 2018; Bommarito et al., 2022; Faivre et al., 2016; Liu et al., 2017; Schoonheim et al., 2022; Shu et al., 2018; Veréb et al., 2021).

A well‐known principle applied in whole‐brain FC analyses is that the human cortex is organized as multiple large‐scale networks which can be reliably assessed from rs‐fMRI data (Thomas Yeo et al., 2011). A previous study has provided evidence that large‐scale FC networks follow a hierarchical arrangement, with primary sensory networks at the bottom, the default mode network (DMN) at the top and the other networks spanning between these two extremes (Margulies et al., 2016). Particularly, the hierarchical organization of the cortex is thought to serve a vital role in supporting increasingly abstract, from lower‐ (perception) to higher‐ (cognition) order, brain functions. This hierarchy is achieved by effectively segregating the information from the immediate environment reaching the primary sensory networks, from the more complex, self‐generated and abstract information processing occurring in higher‐level (integrative) networks (Lanzoni et al., 2020; Margulies et al., 2016; Vos de Wael et al., 2018; Yang et al., 2020). To give a more comprehensive account of the hierarchical axis governing the brain functional organization, Margulies et al. (2016) introduced a computational method based on dimensionality reduction techniques. The method involves the projection of high‐dimensional FC matrix data onto a series of low‐dimensional representations, referred to as functional gradients (FGs), which correspond to the principal components of the FC matrix as obtained via diffusion distance mapping (Coifman et al., 2005). Diffusion distances represent intrinsic geometric quantities enabling the integration of connectivity values within a coherent multiscale structure (Coifman et al., 2005). Indeed, as connectivity measurements include both short‐ (i.e., small‐scale) and long‐ (i.e., large‐scale) range connections, diffusion maps translate these connections into distances enabling the representation of the entire FC structure as a distribution of cortical points in an embedding space. As such, FG values are eigenvalues, and therefore have arbitrary units with no physical meaning, but they represent the relative position of each point/region with respect to the other points/regions along a specific axis (and, therefore, address the similarity of their connectivity profiles), with the two extremes of this axis having the maximum diffusion distance. In their work, Margulies et al. (2016) provided evidence that these FGs are organized along dimensions that describe cortical regions in a hierarchical manner. Thus, estimated FG scores provide a description of the principal axes of spatial FC organization as dictated by the intrinsic similarities among FC patterns. Particularly, FGs, not only represent the dominant patterns of spatial co‐variation in functional organization across the cortex, but are also implicitly ordered in terms of the percentage of (co)variance in FC similarity that they explain (Pang et al., 2023).

FGs have been widely used for investigating brain hierarchical organization in various pathological populations, including migraine (Lee et al., 2023), Alzheimer's disease (Borne et al., 2023; Hu et al., 2022), epilepsy (Lucas et al., 2023; Meng et al., 2021), as well as in healthy individuals (Bethlehem et al., 2020; Cross et al., 2021; Katsumi et al., 2023; Kong et al., 2023; Shen et al., 2023; Vos de Wael et al., 2018; Yang et al., 2020). However, to date, gradients have never been studied in an MS population.

In this study, our primary objective was to investigate alterations in whole‐brain FGs among patients with relapsing–remitting MS and to assess how possible FC anomalies would impact large‐scale networks' hierarchy when compared to individuals from a healthy control (HC) group. We then explored correlations between FG estimates and the most common clinical and neuropsychological scores used in MS patients' assessment. Additionally, using a supervised machine learning framework, we sought to test the performances of FG features as potential imaging markers for predicting IPS of MS patients, as measured by symbol digit modalities test (SDMT) score, that is, the most common metric of cognition in MS, which has been successfully predicted with different FC features in other works (Buyukturkoglu et al., 2021; Lin et al., 2020; Welton et al., 2020).

## 2 METHODS

### 2.1 Participants

A total of 122 patients diagnosed with relapsing–remitting MS (age = 37.6 ± 9.9 years; 84 females) and 97 sex‐ and age‐matched HC subjects (age = 38.1 ± 12.0 years; 56 females) were consecutively recruited at the University of Campania “Luigi Vanvitelli” (Naples, Italy). As for the inclusion criteria, MS patients had to have a diagnosis of MS according to the revised McDonald criteria (Thompson et al., 2018) and a relapsing–remitting phenotype. As for the HC subjects, no T2 hyperintense lesions had to be shown in the MRI scanning. Both MS patients and HCs had to be between 18 and 65 years old, with no history of psychiatric illness. All enrolled participants underwent a neurological evaluation, including the assignment of an expanded disability status scale (EDSS) (Kurtzke, 1983), and a neuropsychological assessment, including the brief repeatable battery of neuropsychological tests (BRB‐N) (Boringa et al., 2001). Among the BRB‐N tests, we specifically focused on the SDMT (Benedict et al., 2017), which is widely used to assess IPS in MS patients (Chiaravalloti & DeLuca, 2008). To interpret the SDMT scores accurately, demographic‐ and education‐adjusted scores were calculated using available normative data based on a sample of 200 healthy Italian adults (Amato et al., 2006), which were then standardized as Z‐scores. All demographic and clinical information are summarized in Table 1.

**TABLE 1:** Demographic and clinical information of study participants. Mean and standard deviation are reported, except for EDSS (median and inter‐quartile range).

	RRMS (*n* = 122)	HC (*n* = 97)	Test statistics	*p*‐Value
Age (years)	37.6 ± 9.9	38.1 ± 12.0	*t* = −0.36	.72
Sex (male:female)	38:84	41:56	*χ* ^2^ = 2.4	.12
Years of education (years)	13.4 ± 3.7	14.0 ± 4.7	*t* = −1.1	.25
Disease duration (years)	10.3 ± 8.7	N/A	N/A	N/A
Lesion load (ml)	4.0 ± 4.1	N/A	N/A	N/A
EDSS	2 (1.5)	N/A	N/A	N/A
BPF	0.71 ± 0.04	0.74 ± 0.02	*t* = −7.8	<.001
SDMT (raw values)	39.8 ± 14.2	N/A	N/A	N/A
SDMT (corrected values)	38.8 ± 13.1	N/A	N/A	N/A
FD (mm)	0.096 ± 0.044	0.105 ± 0.037	*t* = −1.5	.13

Abbreviations: EDSS, expanded disability status scale; FD, framewise displacement; HC, healthy control; RRMS, relapsing remitting multiple sclerosis; SDMT, symbol digit modalities test.

### 2.2 MRI acquisition

All participants underwent the same MRI imaging protocol, which included 3D T1‐weighted (T1w), 2D fluid attenuated inversion recovery (FLAIR) and rs‐fMRI scans, on the same MRI 3 Tesla scanner (Signa HDxt, GE Healthcare, Milwaukee, USA) which was equipped with an eight‐channel head coil. The 3D T1w images were acquired using the following parameters: repetition time (TR) = 6.9 ms; echo time (TE) = 2.8 ms; inversion time (TI) = 650 ms; field of view (FOV) = 256 × 256 mm^2^; flip angle = 8; number of slices = 166; slice thickness = 1.2 mm; in‐plane resolution = 1 mm × 1 mm. The 2D FLAIR images were acquired using the following parameters: TR = 9002 ms; TE = 121 ms; FOV = 512 × 512 mm^2^; flip angle = 90; number of slices = 44; slice thickness = 3 mm; in‐plane resolution = 0.47 mm × 0.47 mm. The rs‐fMRI data were acquired using the following parameters: TR = 1500 ms; TE = 32 ms; FOV = 64 × 64 mm^2^; flip angle = 90; number of slices = 29; slice thickness = 4 mm; in‐plane resolution = 4 mm × 4 mm; number of volumes = 240; scan duration = 6 min. All exams were performed between 8 and 13 a.m. and all subjects were instructed to stay motionless and awake during scanning. Prior to the resting‐state fMRI scan, the subjects were reminded via headphones to remain motionless and awake and to keep their eyes closed for the next 6 min. Ethical approval was received from the local ethical standards committee and written informed consent was obtained from all participants at the time of data acquisition.

### 2.3 Data preprocessing

MRI data were preprocessed using a fully automated pipeline specifically assembled for the serial extraction of FC features from rs‐fMRI data acquired in large samples of subjects including MS patients (De Rosa et al., 2023). In detail, brain tissue segmentation was performed with FreeSurfer v7.1.1 (Fischl, 2012), employing 3D T1w and co‐registered FLAIR scans with the sequence adaptive multimodal segmentation (SAMSEG) (Cerri et al., 2021) procedure to automatically and simultaneously perform whole‐brain tissue segmentation, including white matter (WM), gray matter, cerebrospinal fluid (CSF) volumes, and MS lesion segmentation for lesion load (LL) estimation. Brain atrophy was estimated with the brain parenchymal fraction (BPF) (Rudick et al., 1999), calculated as brain parenchymal volume divided by intracranial volume. Functional MRI data preprocessing was carried out using fMRIPrep v20.2.1 (Esteban et al., 2019). The preprocessing steps included skull stripping, motion correction, slice timing correction, susceptibility distortion correction, and co‐registration of the functional and anatomical scans. To address motion‐related noise, 24 motion‐related predictors were collected, consisting of the 6 head motion parameter time‐series, their first‐order derivatives and the corresponding 12 squared parameter time‐series (Friston et al., 1996). An additional regressor was created from the instantaneous framewise displacement (FD) to account for residual motion‐related spikes (Power et al., 2014; Satterthwaite et al., 2013). These predictors were used as noise regressors in addition to the averaged physiological noise signals from WM and CSF masks. Spatial smoothing was not applied since our FG analysis essentially follows an ROI approach (see Section 2.4 for details), where many rs‐fMRI signals from neighboring voxels are averaged, whether smoothing is applied or not, to produce the time series that represent the ROI activity (Alakörkkö et al., 2017). Moreover, being FC dependent on internode distance, spatial smoothing could introduce distance‐dependent errors into connectivity matrices (Fornito et al., 2013). Finally, the time series were subjected to band‐pass filtering between 0.01 and 0.1 Hz.

### 2.4 FGs computation

The FC matrix was computed for each individual by employing pairwise Pearson's correlation coefficients for the averaged rs‐fMRI time series extracted from 200 distinct brain regions defined using a functional local–global parcellation (Schaefer et al., 2018). According to this parcellation, each region can be seen as a connectome node uniquely assigned to one out of seven large‐scale functional networks as derived from a previous normative rs‐fMRI study (Thomas Yeo et al., 2011). The correlation coefficients underwent Fisher's r‐to‐z transformation prior to FG estimation.

**FIGURE 1:**
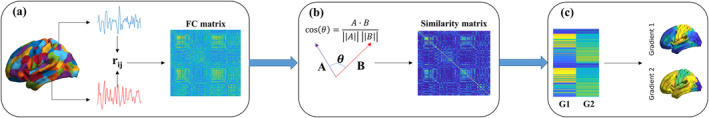
The functional gradient pipeline. (a) Functional time‐series data are extracted from preprocessed functional MRI (fMRI) data and parcellated into 200 cortical regions using the Schaefer atlas. Pearson correlation coefficients are calculated between each pair of the averaged regional time‐series, resulting in a 200 × 200 functional connectivity (FC) matrix. (b) Similarity in connectivity profiles between regions within the FC matrix are estimated based on the difference in angle between pairs of vectors of the FC matrix. Similarity is then expressed through a cosine similarity matrix, representing the shared connectivity patterns between each pair of regions. (c) The application of diffusion map embedding returns a set of principal components ordered by decreasing explained variance. The first two eigenvectors are kept and correspond to the principal and secondary gradient. These eigenvectors are mapped on the cortical surface to visualize the spatial distribution and topology of each gradient.

FGs were computed using BrainSpace, an open‐source tool available at https://github.com/MICA-MNI/BrainSpace (Vos de Wael et al., 2020). Consistently with previous studies (Cross et al., 2021; Meng et al., 2022; Paquola et al., 2019; Yang et al., 2021), a parcel‐to‐parcel affinity matrix was initially calculated to capture the spatial similarity in the FC patterns between brain regions. Specifically, we used a cosine similarity kernel and retained the top 10% of all weighted connections per row. FGs were then obtained through the diffusion map embedding algorithm, which is a robust and computationally efficient nonlinear dimensionality reduction technique (Coifman et al., 2005). With diffusion maps, decreasing eigenvalues reflect the intrinsic ordering of the diffusion process, thereby possible changes in regional FG values would reflect a relative change in the position along the hierarchy established by the corresponding gradient axis. In the resulting embedding space, cortical nodes that exhibit stronger interconnections, either through numerous connections or a few very strong connections are positioned closely together, whereas nodes with weaker or no connections are situated farther apart. For the sake of interpretability, we focused on the first two FGs, in line with previous works (Petersen et al., 2022; Xiao et al., 2023; Zang et al., 2023), as these will describe more variance of the overall FC data and identify the two main cortical hierarchies previously described in literature (Margulies et al., 2016). A gradient template was constructed using a group‐averaged FC matrix from the data sets of all MS and HC individuals. Subsequently, to allow comparisons between subjects, we performed a Procrustes analysis to align the individual participants' cortical gradients with the template (Langs et al., 2015; Vos de Wael et al., 2020). These components, which were initially defined in each subject's native space, were finally mapped to the cortical surface to visualize macroscale transitions in the overall FC patterns. Figure 1 illustrates the processing pipeline to obtain FG estimates from the FC matrix.

We also analyzed gradients at the network level using the seven canonical large‐scale rs‐fMRI networks (Thomas Yeo et al., 2011) with three different gradient metrics. Specifically, in addition to within‐network scores, obtained by averaging the gradient values within each network, we calculated within‐network dispersion and between‐network dispersion (Bethlehem et al., 2020). These metrics were calculated for each participant within the individual, aligned two‐dimensional gradient space, with each axis representing primary and secondary gradient scores. Within‐network dispersion, calculated as the sum of squared Euclidean distances of all network nodes from the network centroid, indicates the degree of differentiation of the single network, thereby, lower (higher) values indicate more (less) uniform FC patterns within that network. Between‐network dispersion, calculated as the Euclidean distance between network centroids, indicates the degree of specificity of the network with respect to the other networks, thereby, lower (higher) values indicate less (more) differentiated FC patterns across different networks.

Finally, a global metric expressing the overall gradient variation was calculated as the standard deviation of each gradient across the whole brain, thereby, higher (lower) values reflect a higher (lower) heterogeneity in the connectivity patterns across all brain regions.

### 2.5 Statistical analysis

To evaluate between‐group differences in regional and network FG scores (i.e., comparing for each region of the Schaefer atlas and for each network of the Yeo parcellation, respectively), we performed statistical analyses on the first two principal components using a multivariate linear model (Hotelling's *T* ^2^ test) implemented in BrainStat, an analysis tool available at https://github.com/MICA-MNI/BrainStat (Larivière et al., 2023). The model aimed to examine differences in FG estimates (response variables) between patient and control groups (explanatory variable), controlling for age, sex, and mean FD. Furthermore, we also used linear models to investigate differences both in terms of within‐ and between‐network dispersion and of whole‐brain gradient variation, controlling for age, sex, and mean FD.

Finally, to study the association of gradient metrics with clinical and neuropsychological scores (i.e., EDSS, disease duration and SDMT) and structural‐derived metrics (i.e., LL and BPF), we conducted a correlation analysis using Spearman's correlation.

For all statistical tests, correction of *p*‐values for multiple comparisons was applied using Bonferroni method.

### 2.6 Machine learning analysis

A supervised machine learning approach was employed to assess the performances of within‐network FG scores as predictive features for SDMT scores. Specifically, we trained a regression model using XGBoost with fivefold nested cross‐validation (Chen & Guestrin, 2016; Tibshirani, 1996; Varma & Simon, 2006). XGBoost is a scalable end‐to‐end tree boosting system that has gained widespread popularity for achieving state‐of‐the‐art performance on many recent machine learning challenges (Chen & Guestrin, 2016). Notably, one of its major advantages lies in the great potential interpretability, due to its recursive tree‐based decision framework (Fratello et al., 2017).

To train, validate and test the XGBoost regression model we adopted the following approach: the entire dataset was partitioned into a training/validation set (80%) and a hold‐out test set (20%). Within the training/validation set, we performed nested cross‐validation, where the inner loop was used to select the optimal hyperparameters configuration for the XGBoost model, while the outer loop was employed for feature selection and model validation. To select the optimal hyperparameters configuration, we employed grid search. To select the best feature set, we used an automated procedure recently applied in other studies (Marzi et al., 2023; Yan et al., 2020). This procedure leverages the importance gain estimated by XGBoost, which reflects the improvement in performance contributed by each feature. We then iteratively trained new XGBoost models using the top n features (*n* = 1, 2, 3, …) in the feature ranking obtained using all the features with the best model selected from the inner loops. The final feature set was chosen based on the best performance in terms of mean absolute error (MAE). The entire nested cross‐validation procedure was repeated 10 times with different fold splits to prevent subject selection bias. To evaluate prediction accuracy, we calculated the average MAE on the validation sets of the outer loops. Significance was determined based on 1000 permutation tests, where participants' SDMT scores were randomly shuffled. Finally, for each repetition, we measured the final model performance on the hold‐out test set.

### 2.7 Experimental setup

Preprocessing of neuroimaging data was carried out on a HP Z6 G4 workstation equipped with two 8‐core Intel (R) Xeon (R) Bronze 3106 @1.70 GHz (for a total of 16 CPU threads) and 128 GB RAM. For each subject, the total processing time was approximately 4 h.

Computational analyses, including FG calculations from neuroimaging data and machine learning, were carried out on an ASUS Pro WS WRX80E‐SAGE SE WIFI workstation equipped with a 64‐core AMD Ryzen Threadripper PRO 5995WX (for a total of 128 CPU threads), a NVIDIA RTX A6000 48 GB GPU and 514 GB RAM. FGs were computed using the BrainSpace toolbox in MATLAB v9.14.0 (R2023a). Statistical and machine learning analyses were carried out with custom‐made codes in Python (v. 3.10). The total computation time for training, validating and testing the model was approximately of 9 h.

## 3 RESULTS

### 3.1 Clinical and demographical characteristics

There were no significant differences in age (*t* = −0.36, *p* = .72), sex ($\chi^{2}=2.4$, *p* = .12) and education (*t* = −1.1, *p* = .25) among the groups (see Table 1). Two (out of 122) MS patients and two (out of 97) HC subjects were left‐handed. No subjects were excluded from the subsequent analyses due to excessive head motion (i.e., mean FD > 0.5 mm). However, six MS patients and one HC subject were excluded due to misregistration of some regions (and therefore missing data from the corresponding nodes of the connectome).

### 3.2 FGs differences

**FIGURE 2:**
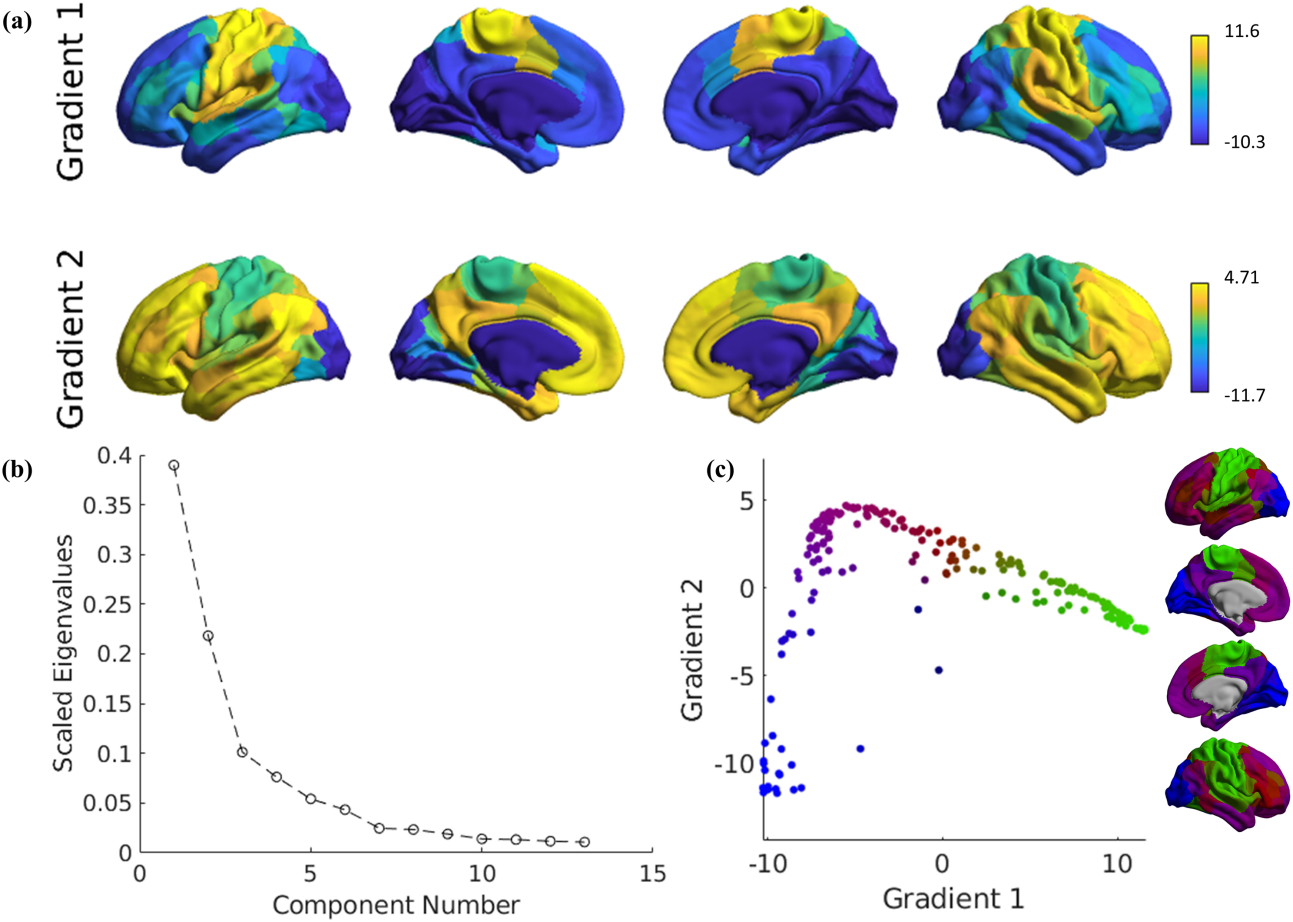
Functional gradients derived from the group‐averaged functional connectivity matrix. (a) Visualization of the functional gradients mapped onto cortical surfaces. The principal gradient (gradient 1) reveals the visual‐sensorimotor axis, while the secondary gradient (gradient 2) reveals the sensory‐transmodal axis. (b) Scree plot of the eigenvalues estimated with the diffusion embedding algorithm. (c) Scatterplot of gradient values in Euclidean space.

The first two FGs explained, respectively, 39.0 and 21.8% of the overall variance in the whole‐brain averaged connectome. Albeit the scree plot of eigenvalues (Figure 2b) indicates a substantial reduction in explained variance after the third principal component, we decided to focus specifically on the first two gradients, as they offer a more straightforward and readily interpretable perspective, as supported by existing literature. Indeed, when projecting the FG values to the cortical surface, the principal gradient highlighted a cortical hierarchy spanning from the visual to the sensorimotor network, while the secondary gradient highlighted a typical (see, e.g., Margulies et al., 2016) sensory‐transmodal axis (Figure 2a,c).

**FIGURE 3:**
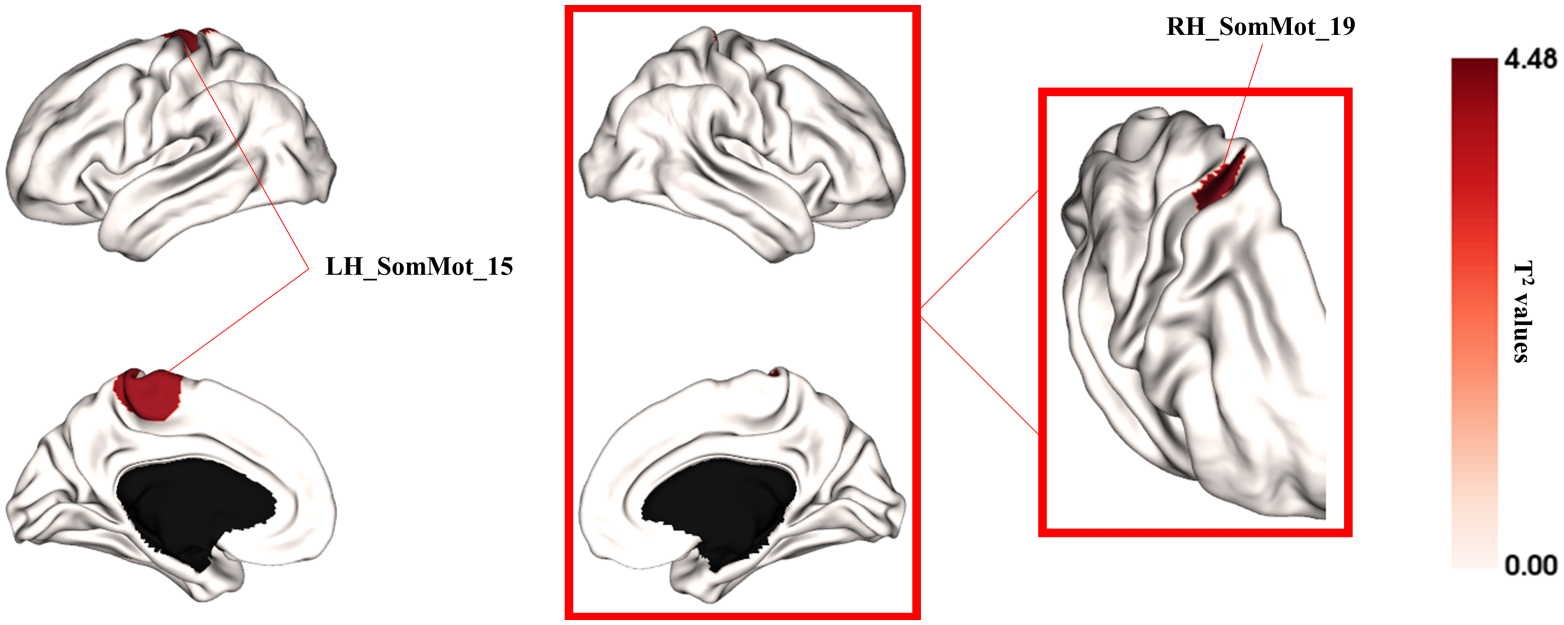
Between‐group differences in the first two gradients between multiple sclerosis (MS) patients and healthy controls (after Bonferroni correction for multiple comparisons, i.e., *p* < .05/200) shown on cortical surface.

The multivariate analysis revealed significant between‐group differences in regional FGs between MS and HC groups. Specifically, we observed significant group effects in two regions of the sensorimotor network after correction for multiple comparisons (*T* ^2^ = 4.13, *p*_bonferroni_ < 0.05; *T* ^2^ = 4.48, *p*_bonferroni_ < 0.05) (Figure 3).

**FIGURE 4:**
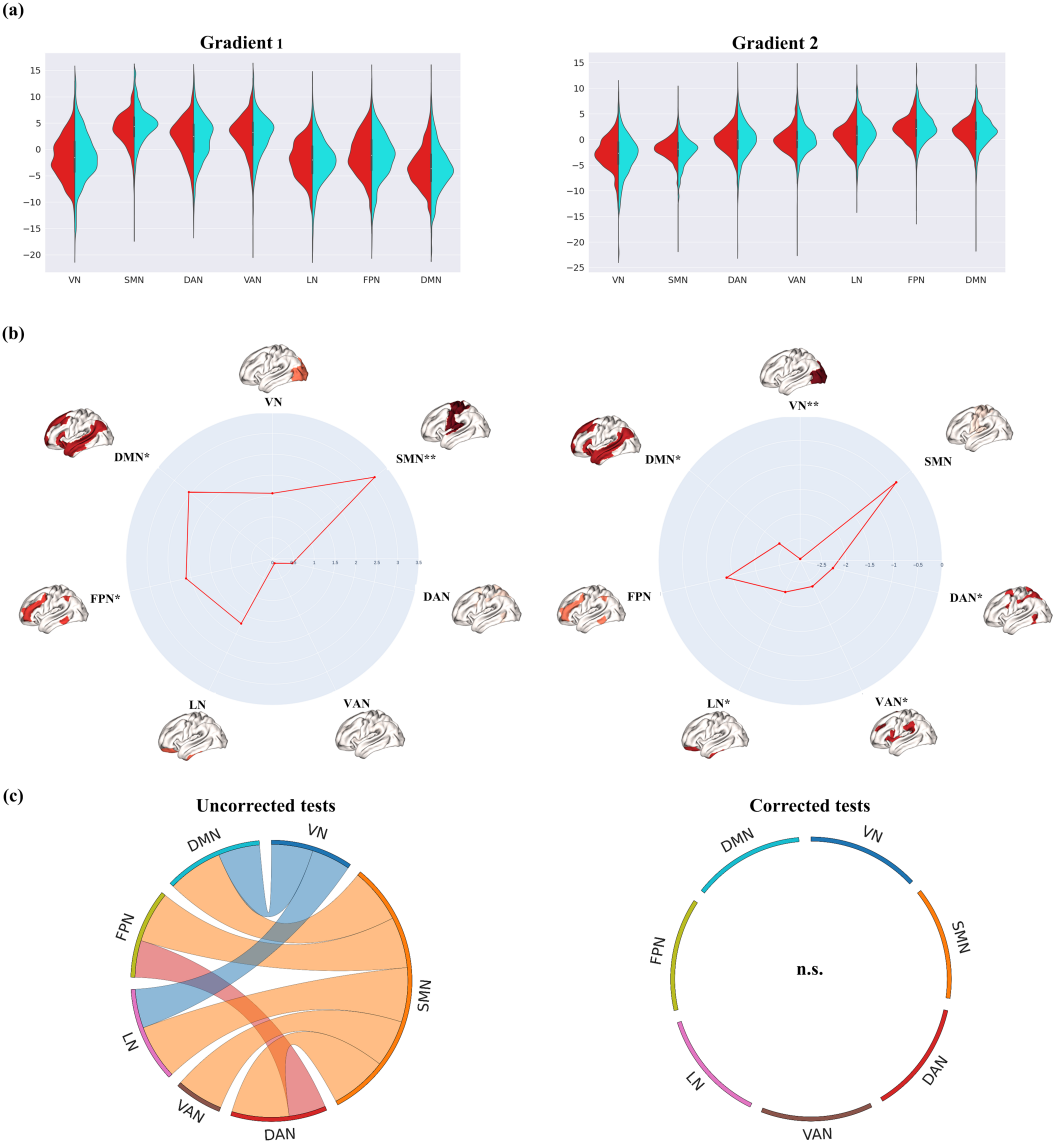
Network‐level comparison. (a) Primary (left) and secondary (right) gradient scores along the seven functional networks. Healthy controls are displayed on the right side of each violin plot (cyan), while multiple sclerosis (MS) patients are displayed on the left (red). (b) Radar plot summarizing the group effect according to the seven functional networks for averaged within‐network gradient values (left) and within‐network dispersion (right). (c) Between‐network dispersion circograms before (left) and after (right) correction for multiple comparisons with Bonferroni. The size of network borders is scaled according to the total effect from that network, with the sensorimotor network being the largest in the left panel as it is most involved in significant between‐network dispersion. *p < .05, uncorrected; **p < .05, Bonferroni corrected. DAN, dorsal attention network; DMN, default mode network; FPN, frontoparietal network; LN, limbic network; SMN, sensorimotor network; VAN, ventral attention network; VN, visual network.

These findings on the regional FG were consistent with network‐level FG comparison: indeed, when the group effect was assessed within the seven intrinsic functional communities, patients with MS exhibited significant alterations of within‐network gradient values in the sensorimotor network (*T* ^2^ = 3.13, *p*_bonferroni_ < 0.05) (Figure 4a,b). Compared to HC subjects, MS patients showed a reduced within‐network dispersion in the visual network (*t* = −2.91, *p*_bonferroni_ < 0.05) (Figure 4b), alongside with reduced between‐network dispersions involving mainly the sensorimotor network, albeit none of them survived multiple comparison correction (*p*_bonferroni_ > 0.05) (Figure 4c).

Finally, even at the global level, MS patients showed a significantly reduced secondary gradient variation across all brain regions (*t* = −2.68, *p*_bonferroni_ < 0.05).

### 3.3 Correlation with MS scores

We explored the potential association between network‐level gradient metrics and disease duration and two other key MS scores (such as EDSS and SDMT) with Spearman's correlation. Among patients with MS, we discovered significant correlations between SDMT scores and the within‐network second gradient values in the limbic network (*p*_bonferroni_ = 0.001, ρ=0.30, CI = [0.12, 0.46]) and in the DMN (*p*_bonferroni_ = 0.001, ρ=0.30, CI = [0.13, 0.46]). No significant correlations were found between disease duration or EDSS and network gradient values. No significant correlations were found between MS scores and network dispersion or variation metrics. No significant correlations were found between any gradient metric and structural metrics (LL and BPF).

### 3.4 Prediction of SDMT scores

**FIGURE 5:**
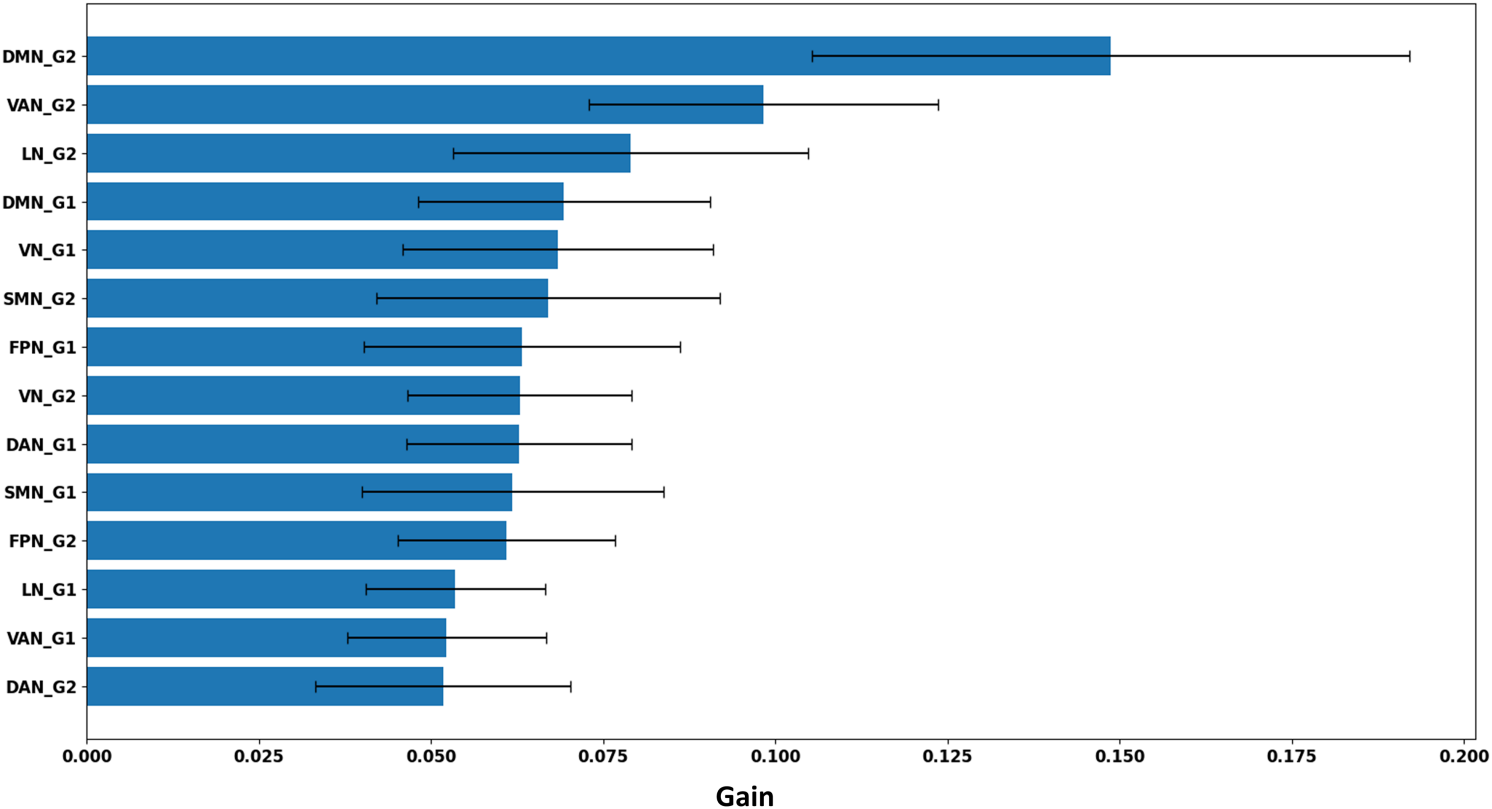
Feature ranking using the XGBoost regression model. Feature importance gain is shown as the averaged values calculated within the validation set across 10 repetitions of the fivefold nested cross‐validation. The black lines with caps indicate the standard deviation. DAN, dorsal attention network; DMN, default mode network; FPN, frontoparietal network; LN, limbic network; SMN, sensorimotor network; VAN, ventral attention network; VN, visual network.

We used supervised machine learning to train an XGBoost regressor to predict SDMT scores using network‐level gradient scores. Across 10 repetitions, secondary gradient values from the default mode, ventral attention and limbic networks (ranked as averaged information gain, Figure 5) mainly contributed to the prediction of SDMT scores and the prediction performance on the validation set was statistically significant when compared against the null distribution as obtained from the permutation tests (MAE = 1.10 ± 0.03, *p*_perm_ < 0.05). Finally, the model has been tested on the hold‐out test set obtaining an average MAE of 1.22 ± 0.07. In a post hoc analysis, Spearman correlation coefficients were calculated between each of the top three ranked features and the predicted SDMT scores. This analysis aimed at evaluating the marginal direction of influence for the top contributing features (i.e., whether lower or higher values lead to lower or higher predicted scores). Across 10 repetitions of the nested cross‐validation, Spearman's *ρ* consistently exhibited a positive trend for the default mode and limbic networks (*ρ* = 0.57 ± 0.07 and *ρ* = 0.37 ± 0.09, respectively). Conversely, the correlation was consistently negative for the ventral attention network (*ρ* = −0.31 ± 0.11).

## 4 DISCUSSION

Cortical hierarchy represents a fundamental organizational principle of the human brain connectivity. Several previous works highlighted network‐level connectivity alterations in MS disease (Bisecco et al., 2018; Bommarito et al., 2022; Faivre et al., 2016; Liu et al., 2017; Schoonheim et al., 2022; Shu et al., 2018; Veréb et al., 2021) but whether and how network hierarchy changes in MS has remained elusive. To address this gap, we sought to investigate alterations in whole‐brain FC in patients with relapsing–remitting MS, focusing on the hierarchical axes of cortical organization by employing a nonlinear dimensionality reduction technique to generate low‐dimensional representations of FC matrices, commonly referred to as FGs (Bethlehem et al., 2020; Margulies et al., 2016; Park et al., 2021; Vos de Wael et al., 2020).

Preliminarily, we observed that the order of the first two FG patterns was inverted with respect to previous findings. In particular, the principal gradient was anchored between the visual and sensorimotor networks and this closely resembled the secondary gradient previously identified in (Margulies et al., 2016). Instead, the secondary gradient showed a gradual axis of connectivity variation, with the visual network on one end and the DMN on the other, with intermediary networks in between, similar to the principal gradient previously identified in (Margulies et al., 2016). This discrepancy has been previously observed and attributed to different factors, including preprocessing strategies (Hong et al., 2020; Knodt et al., 2023), age range of the studied population (Bethlehem et al., 2020; Hu et al., 2022), and sample size (Zang et al., 2023), whereas it should be independent from parcellation resolution (Vos de Wael et al., 2020). In this study, participants were relatively young, thus suggesting that age should not significantly affect FGs; on the other hand, the sample size was not as small as in other works reporting the canonical FGs (Lee et al., 2023; Zhang et al., 2022). Therefore, it is reasonable to hypothesize an influence of the rs‐fMRI data preprocessing strategy behind the inverted FGs. However, a robust and validated preprocessing pipeline (Esteban et al., 2019) was used for the present study and a systematic investigation on the impact of different preprocessing strategies on the order of FGs is beyond the scope of this work.

We investigated FGs at three different scales: regional‐level (regional gradient scores), network‐level (within‐network gradient score and dispersion metrics) and global‐level (gradient variation). As we used the functional local to global parcellation by Schaefer et al. (2018), we were guaranteed that each parcel was also identically assigned to one (and only one) of the seven large‐scale networks. Hence, we assigned a gradient score to each network. While this metric is not a direct measure of cortical hierarchy, it provides an alternative perspective on the brain functional organization and on whether this organization varies between healthy subjects and MS patients. Indeed, when compared between two groups, within‐network gradient scores provide insight into whether a network, on average and on a broader scale than cortical FGs, occupies a different position in the brain hierarchy. In essence, the seven pre‐defined networks served as geometrical constraints to derive a network gradient metric built upon the data driven method. Our results revealed atypical hierarchical organization in the sensorimotor areas of MS patients compared to the control group, both at regional‐ and network‐level. Albeit only descriptively, we could show how these changes in the gradient architecture seem to have deeply impacted the macroscale network organization, as also suggested by the reduced between‐network dispersion of the sensorimotor network, which clearly indicated a worse differentiation from the other networks. Moreover, we found that the visual network in MS patients was significantly less dispersed compared to HC subjects. Finally, the secondary gradient (i.e., the sensory‐transmodal gradient) was significantly more compressed (meaning that it showed reduced variation) in the MS group.

Through correlation analyses, we identified significant associations between the second gradient values in the limbic and DMNs and the SDMT scores within the MS group. These findings provide valuable insights into how FC hierarchy alterations relate to IPS in MS. To further explore the potential clinical significance of FGs and their predictive power for cognitive status, we leveraged supervised machine learning. Specifically, we employed an XGBoost regression model and implemented a rigorous nested cross‐validation strategy for hyperparameters tuning and feature selection in the training/validation set. Moreover, since these decisions may vary depending on how the training/validation data are split in each fold, we repeated this process 10 times. Finally, the selected best model was evaluated on a hold‐out test set. Our findings from supervised machine learning revealed that the within‐network FG score in the DMN play a crucial role in predicting SDMT scores, that is, IPS, of MS patients.

Overall, the present study provides novel evidence that the sensorimotor hierarchy is significantly altered in patients with MS compared to HCs. This observation reflects disrupted segregation between the sensorimotor and the remaining networks, as suggested by the altered between‐network dispersion. Dysfunction in the sensorimotor network has been widely documented for patients with MS in previous studies (Carotenuto et al., 2022; Jaeger et al., 2019; Rocca et al., 2010; Schiavi et al., 2020; Schoonheim et al., 2014; Strik et al., 2021). In particular, the work of Strik et al. (2021) highlighted the clinical relevance of functional changes in the sensorimotor network, which were showed to occur independently from structural damage. Consistent with these results, the current study revealed a different aspect of such alterations in the sensorimotor network, possibly suggesting that the pathological interaction between sensory and cognitive processes in MS would be reflected also by an abnormal communication between the sensorimotor and the high order cognitive systems along the hierarchical organization of the brain, and not necessarily by the structural damage (e.g., LL) or atrophy (e.g., BPF). This disrupted integration process has been further characterized in this study by the overall compression of the sensory‐transmodal gradient, which reflects diminished separation between sensory systems (e.g., visual and sensory regions), responsible for the immediate environment processing, and transmodal cognitive systems (e.g. frontoparietal and default mode regions), that support complex cognitive functions (Dong et al., 2023).

The range of individual networks across gradients was also altered in MS patients, with reduced within‐network dispersion in the visual network, thus suggesting more homogeneous FC patterns. Notably, previous studies using rs‐fMRI in MS have already observed FC anomalies in visual network regions (Backner et al., 2022; Gallo et al., 2012).

We have also showed a significant correlation between SDMT scores and the within‐network secondary gradient values in the limbic and DMNs, implying an association in our cohort of patients between the variance in FG secondary patterns across these two networks and SDMT scores. These findings are consistent with previous studies that applied different frameworks for the FC analysis of rs‐fMRI data (e.g., comparing FC maps or independent components or applying graph theory metrics to the connectome). Particularly, the limbic network is known to be involved in memory, emotional processing and reward‐related functions (Cao et al., 2021; Rolls, 2019) and its dysfunction in MS patients has been previously reported. Bisecco et al. (2020) found a significant association in certain regions of the limbic network between FC and social cognition, which is a strong correlate of SDMT (Bisecco et al., 2020; Raimo et al., 2017). The DMN is another crucial brain network implicated in cognitive dysfunction in MS (Bommarito et al., 2022; Bonavita et al., 2011; Bonavita et al., 2017; Eijlers et al., 2017; Rocca et al., 2018), and particularly its association with IPS has been also reported (Has Silemek et al., 2020; van Geest et al., 2018). No significant correlations were found with nonspecific measures, such as disease duration and EDSS scores.

Our findings were further reinforced by the integration of a machine learning framework designed to predict SDMT performance using within‐network gradient scores. We decided to restrict the prediction analysis to SDMT since EDSS, the most common measure to evaluate disease‐related disability, as a general (multi‐domain) score, has been showed to be less sensitive to FC changes induced by large‐scale networks remodeling (Cipriano et al., 2023). Indeed, it has been showed that SDMT is the most reliable and sensitive cognitive measure in MS and is commonly regarded as the best predictor of cognitive status (Benedict et al., 2017; Van Schependom et al., 2014). Moreover, several previous works showed that FC changes were significantly associated with SDMT (Buyukturkoglu et al., 2021; Lin et al., 2020; Welton et al., 2020). Here, we adopted the hold‐out method to split the entire dataset into a training/validation set (80%) and test set (20%). Within the training/validation set, a fivefold nested CV scheme was applied, enabling simultaneous hyperparameters optimization and feature selection. It is noteworthy that, to the best of our knowledge, only Marzi et al. (2023) applied such a similar rigorous split of training/validation and test sets to predict a cognitive score in an MS population, but no other works have focused on prediction relying exclusively on FC features. Results revealed that secondary FGs from the default mode, ventral attention and limbic networks provided the top three FG features contributing to the prediction of individual SDMT scores (thereby suggesting that changes in these features have a larger impact on predictions), with the DMN, in particular, emerging as an important potential predictor of cognitive status in MS patients. Notably, FC alterations within the DMN have been previously recognized to provide a potential marker for cognitive impairment (Bonavita et al., 2017; Eijlers et al., 2017). Indeed, the DMN plays a critical role as a central hub for processing and integrating transmodal information, thus likely contributing to IPS dysfunction. We further investigated how single features affect predictions by examining the direction of their influence. Results revealed that higher secondary gradient values within limbic and DMNs, alongside with lower values within ventral attention network, are associated with higher predicted SDMT scores. Notably, while the former (positive) correlations aligned with the prior correlation analysis, the XGBoost predictive model additionally highlighted the role of the ventral attention network secondary gradient values in SDMT score prediction. The ventral attention network plays a critical role in regulating networks involved in cognition (Menon & Uddin, 2010). Moreover, FC changes in ventral attention network have been extensively reported in MS studies (Huang et al., 2019; Rocca et al., 2012) and a recent study from Huiskamp et al. (2021) demonstrated a negative correlation with cognitive performances (Huiskamp et al., 2021). Altogether, these findings would allow indicating the degree of segregation between the ventral attention network and higher‐order networks (i.e., limbic and DMNs) as an important determinant of cognitive status prediction. More specifically, higher segregation seems to be more characteristic of MS patients with higher SDMT scores.

Overall, this work presents compelling evidence that FGs, by addressing complementary FC aspects related to the hierarchical organization of typical FC networks, can serve as a reliable tool for analyzing rs‐fMRI data in the context of MS research. More importantly, network hierarchy provides a novel perspective for exploring the intricate neuro‐mechanisms underpinning MS. The added value of investigating macroscale functional anomalies with FGs lies in its ability to capture the whole‐brain organizational principles in a single manifold, overcoming the challenges associated with handling high‐dimensional FC matrices and offering more plausible and valuable insights into the integrated nature of neural processing. This seems to hold great potential and promise for shedding more light on the brain mechanisms underlying the switch between function and dysfunction.

Our study has two potential limitations. First, lesion segmentation masks (and the derived LL) for a large cohort of MS patients were here obtained via a fully automated segmentation method (SAMSEG) without manual review. In fact, the SAMSEG method incorporates a sufficiently general model for WM lesions which has been previously validated with, and shown to adapt well to, several data sets acquired with different scanners and imaging protocols (Cerri et al., 2021). Nonetheless, most currently available fully automated methods for lesion segmentation may include some degree of error which would be only addressed by careful visual inspection followed by expert manual review of each individual mask, thereby the results of the structural analysis involving estimated LL (i.e., correlation with within‐network FG values) should be taken with more caution. For this reason, we also did not further explore whether the location, shape or type of the estimated lesions had an impact on FGs. Second, to limit the dimensionality of the FC matrix, we chose a brain functional parcellation for FG computation at a scale of 200 nodes even if we could presume that by increasing spatial resolution the delineation of FC gradients might have resulted more pronounced. However, this would be certainly true and effective for the purposes of the present study only when using anatomically‐informed parcellations (see, e.g., Vos de Wael et al., 2020) whereas we expressly chose a functionally‐informed parcellation, that is, the local–global parcellation originally derived by Schaefer et al. (2018), also because a high correspondence between Mesulam's classic scheme of cortical hierarchy (Mesulam, 1998) and FGs, was highlighted for this specific brain parcellation, even at a coarser scale (i.e., 300 nodes or less). Thus, future studies focusing on FGs in MS should assess the possible association with anatomy and structure in general and with lesions in particular (including location and type) with more resolution (e.g., at a higher scale) and precision, thus providing more information on how the neuroinflammatory processes of the MS disease contribute to, or interact with, the observed alterations in the cortical hierarchy.

In conclusion, in this study, we investigated the alterations in FGs in patients with MS compared to HCs. By exploring alterations in the whole‐brain cortical hierarchy, we expanded previous research that primarily focused on disruptions of network connectivity. We complemented these findings by conducting a machine learning prediction analysis. Our work may provide valuable insights into the comprehensive understanding of whole‐brain alterations in MS patients, particularly on network hierarchy anomalies.

## FUNDING INFORMATION

This work was supported by #NEXTGENERATIONEU (NGEU) and funded by the Ministry of University and Research (MUR), National Recovery and Resilience Plan (NRRP), project MNESYS (PE0000006)—A multiscale integrated approach to the study of the nervous system in health and disease (DN. 1553 11.10.2022).

## CONFLICT OF INTEREST STATEMENT

The authors declare that they have no known competing financial interests that could have appeared to influence the work reported in this paper.

## Data Availability

Data used in the current study are available from the corresponding author upon reasonable request. The codes used for the analyses are available at https://github.com/apderosa/GradientsMS.
